# Complete Genome Sequences of Zika Virus Strains Isolated from the Blood of Patients in Thailand in 2014 and the Philippines in 2012

**DOI:** 10.1128/genomeA.00359-16

**Published:** 2016-05-12

**Authors:** D. W. Ellison, J. T. Ladner, R. Buathong, M. T. Alera, M. R. Wiley, L. Hermann, W. Rutvisuttinunt, C. Klungthong, P. Chinnawirotpisan, W. Manasatienkij, M. C. Melendrez, I. Maljkovic Berry, B. Thaisomboonsuk, P. Ong-ajchaowlerd, W. Kaneechit, J. M. Velasco, I. A. Tac-An, D. Villa, C. B. Lago, V. G. Roque, T. Plipat, A. Nisalak, A. Srikiatkhachorn, S. Fernandez, I. K. Yoon, A. D. Haddow, G. F. Palacios, R. G. Jarman, L. R. Macareo

**Affiliations:** a Armed Forces Research Institute of Medical Sciences, Bangkok, Thailand; b Center for Genome Sciences, United States Army Medical Research Institute of Infectious Diseases, Fort Detrick, Frederick, Maryland, USA; c Department of Disease Control, Bureau of Epidemiology, Ministry of Public Health, Nonthaburi, Thailand; d Walter Reed Army Institute of Research, Silver Spring, Maryland, USA; e Cebu City Health Department, Cebu City, Philippines; f Department of Health, Manila, Philippines; g University of Massachusetts Medical School, Worcester, Massachusetts, USA; h United States Army Medical Materiel Development Activity, Frederick, Maryland, USA; i International Vaccine Institute, Seoul, Republic of Korea

## Abstract

Here, we present the complete genome sequences of two Zika virus (ZIKV) strains, Zika virus/*Homo sapiens*-tc/THA/2014/SV0127-14 and Zika virus/*H. sapiens*-tc/PHL/2012/CPC-0740, isolated from the blood of patients collected in Thailand, 2014, and the Philippines, 2012, respectively. Sequencing and phylogenetic analysis showed that both strains belong to the Asian lineage.

## GENOME ANNOUNCEMENT

Zika virus (ZIKV) has garnered worldwide attention, as researchers have linked the virus to an increase in microcephaly cases during the current outbreak in South America (1). Once thought to cause mild infections, ZIKV is now the subject of intensive worldwide research collaborations and efforts. ZIKV is a single-stranded positive-sense RNA arbovirus and a member of the *Flaviviridae* family, which includes dengue, yellow fever, St. Louis encephalitis, Japanese encephalitis, and West Nile viruses (2). The ZIKV genome, approximately 11 kb in length, is similar in its arrangement to other members of *Flaviviridae* containing 5′ and 3′ untranslated regions (UTRs) flanking a single open reading frame (ORF). The 5′ and 3′ UTRs are thought to be important for host interaction, viral replication, and pathogenicity. The ORF codes for three structural proteins: the capsid, premembrane/membrane and envelope, and seven nonstructural proteins, which are responsible for viral replication and assembly (3). Previous phylogenetic analysis based on the nucleotide sequences of ZIKV indicated there to be two major lineages, African and Asian (2).

These two ZIKV isolates were obtained by intrathoracic inoculation of ZIKV real-time reverse transcription-PCR (RT-PCR)-positive patient serum samples into *Toxorhynchites splendens* mosquitoes, followed by inoculation of mosquito-derived C6/36 cells (4, 5). Viral RNA was extracted using the Direct-zol RNA extraction kit (Zymo Research), converted to cDNA using SuperScript III (Invitrogen), and amplified using sequence-independent single-primer amplification (6) combined with primers for rapid amplification of cDNA ends (7). Sequencing libraries were constructed using the PrepX ILM 32 DNA library kit (WaferGen) and sequenced by using the Illumina NextSeq platform (2 × 151 bp). Adaptors and primers were clipped using Cutadapt version 1.21 (8), and low-quality reads/bases were filtered using Prinseq-lite version 0.20.4 (9) ZIKV consensus genomes were assembled using Ray Meta (10), Bowtie2 version 2.0.6 (11), and SAMtools version 0.1.18 (12).

The complete genome sequence, with a total length of 10,807 nucleotides (nt), containing the 5′ (107 nt) and 3′ (428 nt) UTRs and one ORF (10,272 nt), were obtained from both isolates (13). Our sequences and other ZIKV sequences from GenBank were used in maximum-likelihood phylogenetic analysis by using PhyML 3.1 (14) with a general time reversible (GTR)+G model (–lnL 33,630.580); the tree revealed that these two isolates belong to the Asian lineage and are closely related to Brazilian isolates. This confirms our previous analyses based on nonstructural protein 5 (NS5) genes of these two isolates (4, 5). The Asian lineage isolates are 95 to 98% identical on the nucleotide level and 98 to 99% identical on the amino acid level. Nevertheless, some substitutions were found within primer/probe binding sites in the genomes of these two isolate sequences. The two mismatches were found in the Zika virus/*Homo sapiens*-tc/PHL/2012/CPC-0740 genome for primer 835 (residue 1/23) and for probe 1107 (residue 1/31) binding sites (15). There are two mismatches found in the Zika virus/*H. sapiens*-tc/THA/2014/SV0127-14 genome for primer 911c (residue 21/22) and for the probe 1107 (residue 19/31) binding site (15). Further ZIKV genome studies and comparisons will not only elucidate factors involved in the virulence and pathogenicity of ZIKV but will also lend insight into the evolution of this virus and help with vaccine design.

### Nucleotide sequence accession numbers.

The assembled complete genome sequences of the Zika virus/*H. sapiens*-tc/THA/2014/SV0127-14 and Zika virus/*H. sapiens*-tc/PHL/2012/CPC-0740 isolates were submitted to GenBank under the accession numbers KU681081 and KU681082, respectively.
